# A novel technique for measuring variations in DNA copy-number: competitive genomic polymerase chain reaction

**DOI:** 10.1186/1471-2164-8-206

**Published:** 2007-07-02

**Authors:** Kyoko Iwao-Koizumi, Kazunori Maekawa, Yohko Nakamura, Sakae Saito, Shoko Kawamoto, Akira Nakagawara, Kikuya Kato

**Affiliations:** 1 Research Institute, Osaka Medical Center for Cancer and Cardiovascular Diseases, 1-3-2 Nakamichi, Higashinari-ku, Osaka 537-8511, Japan; 2 Division of Biochemistry, Chiba Cancer Center Research Institute, Chiba 260-8717, Japan

## Abstract

**Background:**

Changes in genomic copy number occur in many human diseases including cancer. Characterization of these changes is important for both basic understanding and diagnosis of these diseases. Microarrays have recently become the standard technique and are commercially available. However, it is useful to have an affordable technique to complement them.

**Results:**

We describe a novel polymerase chain reaction (PCR)-based technique, termed competitive genomic PCR (CGP). The main characteristic of CGP is that different adaptors are added to the sample and control genomic DNAs after appropriate restriction enzyme digestion. These adaptor-supplemented DNAs are subjected to competitive PCR using an adaptor-primer and a locus-specific primer. The amplified products are then separated according to size differences between the adaptors. CGP eliminates the tedious steps inherent in quantitative PCR and achieves moderate throughput. Assays with different X chromosome numbers showed that it can provide accurate quantification. High-resolution analysis of neuroblastoma cell lines around the MYCN locus revealed novel junctions for amplification, which were not detected by a commercial array.

**Conclusion:**

CGP is a moderate throughput technique for analyzing changes in genomic copy numbers. Because CGP can measure any genomic locus using PCR primers, it is especially useful for detailed analysis of a genomic region of interest.

## Background

Variations in DNA copy number occur in many diseases such as Down, Prader-Willi, Turner and Angelman syndromes, as well as in cancer. In particular, a loss or gain of DNA copy number is frequently observed in cancer, affecting (respectively) tumor suppressor genes and oncogenes. Techniques that detect abnormalities in DNA copy number are therefore useful for studying the associations between DNA aberrations and disease phenotype and for locating critical genes.

Comparative genomic hybridization (CGH) was developed for genome-wide analysis of DNA copy number and is based on two-color fluorescence *in situ *hybridization (FISH) [1]. In CGH, differentially-labeled total genomic DNAs from a 'test' and a 'reference' cell population are co-hybridized to normal metaphase chromosomes, using blocking DNA to suppress signals from repetitive sequences. The resulting fluorescence intensity ratio at a location on the 'cytogenetic map' of the chromosomes is approximately proportional to the ratio of copy numbers between the corresponding DNA sequences in the test and reference genomes. However, the use of metaphase chromosomes limits the detection of events involving small regions (less than 20 Mb) of the genome, i.e. the resolution of closely spaced aberrations, making it difficult to assign their genomic locus. Moreover, CGH results must be adjusted for biases in the correlation of the heteroscedastic data distribution in a two-color FISH [2], although use of the "dye swap" method substantially reduces this problem.

Recently, a DNA microarray-based CGH was developed [3-5]. In addition, oligonucleotide arrays for detecting SNP have been used to analyze genomic copy numbers [6]. These microarray-based methods have higher resolution than FISH-based CGH, and the recent availability of commercial products has increased the popularity of this technique. However, because currently available arrays have gaps between probes, some regions are not available for analysis. It is important to have a technique for making detailed measurements of DNA copy number in regions of interest.

In this report, we describe a new technique for measuring changes in gene copy number to meet these needs. Polymerase chain reaction (PCR) is an alternative technique for quantifying genome copy number. Although several reports have used PCR to measure DNA copy numbers [7-9], it has not gained broad popularity because careful calibration is required for accurate quantification. In the new PCR-based technique described here, competitive genomic PCR (CGP), competitive PCR is performed using restricted genomic DNA ligated to specific adaptors as a template. Different adaptors are added to the test and control samples and the test-to-reference ratio is determined by quantifying the amplified products fractionated by gel electrophoresis. CGP does not require preliminary calibration experiments, considerably improving the throughput and rapidity of the experimental process. This technique complements hybridization-based techniques for CGH and can easily serve as an alternative to FISH or array-based CGH, especially for high-resolution analysis of a particular chromosome region.

## Results

### Outline of the CGP assay

**Figure 1:**
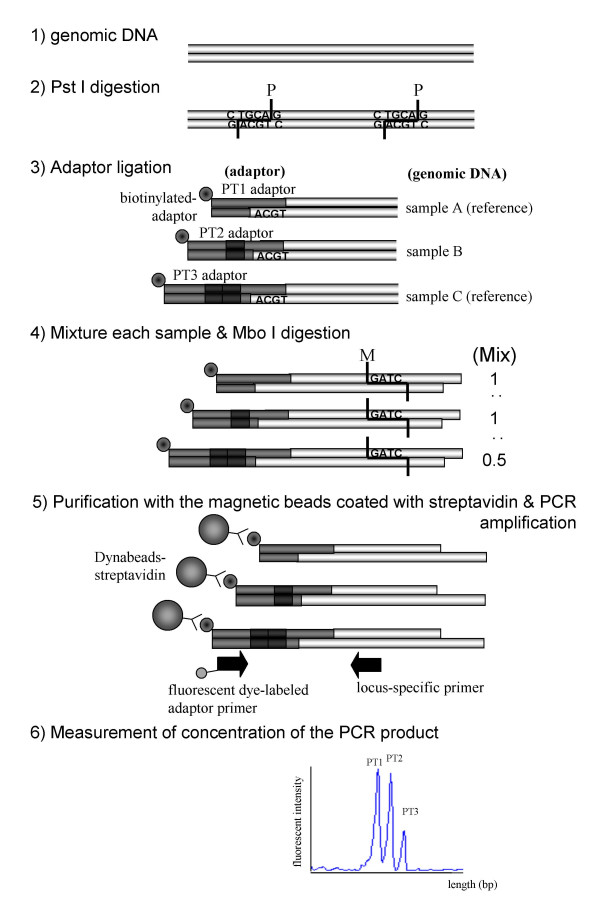
Outline of the CGP assay. Schematic representation of the CGP assay for quantification using PstI and MboI as restriction enzymes.

The CGP assay comprises several enzymatic steps to produce a PCR template containing genomic DNA ligated to specific adaptors. A scheme of the method is presented in Fig. 1. First, double-strand genomic DNA was digested with the restriction endonuclease Pstl, which cleaves at CTGCAG. The first 5'-biotinylated adaptor (PT1) was then ligated to DNA sample A, the adaptor (PT2) to sample B and the third (PT3) to sample C (Fig. 1). The three adaptors shared a common sequence and had a cohesive end complementary to the PstI restriction site; however, the second adaptor was 3 bases longer than the first and the third was 3 bases longer than the second (these extra bases were located between the common sequence and the overhang). After ligation, we mixed the three DNA samples; the first and the third adaptors were both ligated with Fmix DNA as a reference (see Materials and Methods), but in different quantities (one equivalent or one half equivalent, respectively), while the second was used for the test sample (one equivalent). The samples were digested with the restriction endonuclease MboI, which recognizes the 4 bp cleavage site GATC. Adaptor-supplemented genomic DNA fragments were recovered using streptavidin-coated paramagnetic beads and were PCR-amplified using an adaptor-specific and a locus-specific primer complementary to a sequence near the PstI recognition site. MboI digestion and subsequent purification by the beads removes most of genomic DNA, which may result in artifactual amplification. For detection, the adaptor-primer was labeled with a fluorescent dye. The amplified fragments were separated by denaturing polyacrylamide gel electrophoresis and quantified with an automated sequencer.

**Figure 2:**
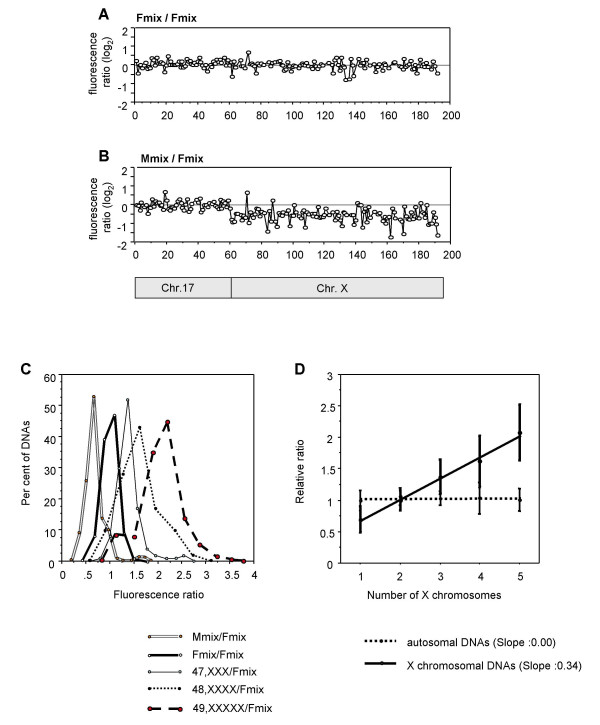
**A, B. **Validation of the CGP assay using Fmix vs. Fmix (**A**), or Fmix vs. Mmix (**B**). The horizontal axis displays 132 loci of chromosome X and 60 loci of chromosome 17. The vertical axis represents test/reference log_2 _fluorescence ratio of each locus. **C, D. **Sensitivity of CGP for detecting a change in low-level copy number. CGP was performed to determine the copy number of loci in the chromosome X. Fmix DNA (46, XX) was used as a reference DNA for all reactions. The distribution of test/reference fluorescence ratios for the different test samples of chromosome X DNA (**C**). Vertical axis, the percentage of DNAs; horizontal axis, test/reference fluorescence ratio. The doublet line, bold line, solid line, dotted line and dashed line indicate the Mmix/Fmix, Fmix/Fmix, 47, XXX/Fmix, 48, XXXX/Fmix and 49, XXXXX/Fmix fluorescence ratios, respectively. The mean relative ratios of autosomal DNAs (dotted line) and chromosome X DNA (solid line) from each experiment versus the number of X chromosomes (**D**). Mean (± 1 s.d.) fluorescence ratios of chromosome X DNA were as follows; XY versus XX, 0.69 (0.49–0.89); XX versus XX, 1.00 (0.84–1.15); XXX versus XX, 1.37 (1.10–1.64); XXXX versus XX, 1.62 (1.21–2.02); XXXXX versus XX, 2.08 (1.63–2.52). A dotted line for chromosome X and a solid line for autosomal mean fluorescence ratios were fitted using standard regression analysis.

Using this procedure, we tested the CGP assay using Fmix and Mmix DNAs (see Materials and Methods) as references for 132 loci on chromosome X and 60 loci on chromosome 17. When we used Fmix DNA for both the test and reference samples, the test/reference fluorescence ratios measured for loci on chromosome X were tightly distributed around a log_2 _value of 0 (Fig. 2A). In contrast, when we compared genomic DNA from Mmix (46, XY) with Fmix (46, XX), the distribution of fluorescence ratios for chromosome X loci was shifted and approached a mean log_2 _value of -1, while the ratios for loci on chromosome 17 remained at a log_2 _value of 0. These differences reflect the absence of the single-copy chromosome X from the control male samples (Fig. 2B). The standard deviation of the log_2 _values was 0.27 excluding outliers, and 0.31 including outliers.

### Design of locus-specific primers for the CGP assay

In the CGP assay, it is important that each primer binds to a single locus in the genome. In addition, because CGP is used to study genetic changes in cancer, the loci used for quantification should be located near genes that are commonly expressed in cancer tissues. To fulfill these requirements we adopted the following strategy.

We previously performed gene expression profiling on more than a thousand cancer tissues by adaptor-tagged competitive PCR (ATAC-PCR) [10-14] for the Cancer Gene Expression Database (CGED) [15,16]. For each cancer type we performed middle-scale EST sequencing (the number of tags usually exceeded 5,000), and selected genes for assay among the expressed genes. It should be noted that our ESTs correspond to an mRNA region with an MboI site closest to the 3' end. The number of ESTs sequenced to date is 18,042. We used this EST collection to construct the CGP primer library.

**Figure 3:**
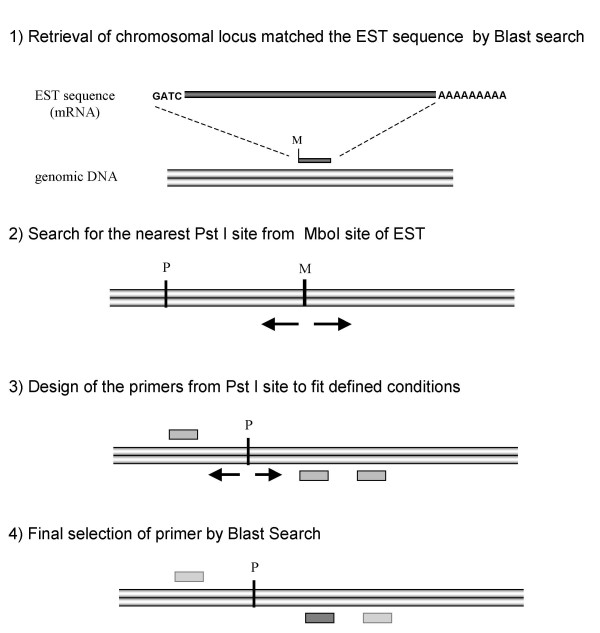
Outline of locus-specific primer design. 'M' and 'P' indicate the MboI and PstI restriction sites, respectively.

The procedure for designing the locus-specific primers is presented schematically in Fig. 3. First, all the ESTs in the collections were compared with the human genome by a BLAST search of the NCBI database [17]. When an EST matched a single chromosomal locus, we isolated it together with the surrounding 10 kbp of genomic DNA. Second, we searched for PstI restriction sites in the region surrounding the MboI site of the EST. We designed primers that were 15–65 bases downstream or upstream from the PstI site and had a melting temperature between 56 and 64°C. Using the dbSNP database [18], we eliminated primer sequences or PstI sites containing SNPs and primers in which a MboI restriction site was present between the PstI site and the locus-specific primer sequence. Finally, the primers were screened using a BLAST search in Ensembl [19]; we selected only those that matched a single chromosomal locus. The primer sequences for the whole genome are supplied as the additional data file 1.

### Detection of changes in low-level DNA copy number

To test the sensitivity of CGP in detecting changes in single copy numbers, we used genomic DNA from cell lines with varying numbers of X chromosomes (to simulate distinct levels of gene amplification and deletion) and compared 132 loci of chromosome X with 60 loci of chromosome 17.

When we compared two Fmix samples used as both test and reference DNA, the test/reference fluorescence ratios measured for chromosome X loci were tightly distributed around a mean value of 1 (Fig. 2C). In contrast, when we compared genomic DNA from Mmix (46, XY) with Fmix (46, XX), the distribution of fluorescence ratios for chromosome X loci was shifted to a mean value of 0.69 (expected 0.5; Fig. 2C, D). Similarly, when we compared genomic DNAs from 47, XXX 48, XXXX and 49, XXXXX cell lines using Fmix DNA, the distribution of fluorescence ratios for chromosome X loci was shifted to mean log_2 _values of 1.37, 1.62 and 2.08, respectively (expected 1.5, 2.0, 2.5; Fig. 2C, D), reflecting the increase in chromosome X DNA copy-number. The mean fluorescence ratios for chromosome X loci obtained in the different experiments displayed a tight linear fit (Fig. 2D), with a correlation coefficient of 0.996. This demonstrates that the fluorescence ratios were linearly proportional to DNA copy number even with a low-level gene-amplification or single-copy deletion.

### Detection of v-myc myelocytomatosis viral-related oncogene (MYCN) amplification and analysis of the chromosome breakpoints

To characterize the ability of CGP to measure differences in copy number in genomic DNA, we evaluated regional amplification in neuroblastoma-derived cell lines.

**Figure 4:**
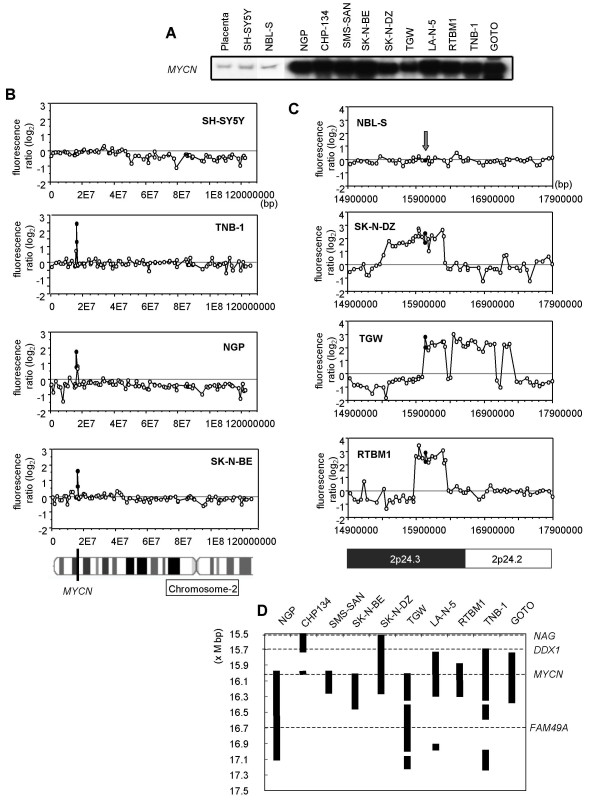
Detection of MYCN gene amplification in neuroblastoma cell lines in CGP. **A. **Confirmation of *MYCN *amplification in neuroblastoma cell lines by Southern hybridization. **B. **CGP analysis of neuroblastoma cell lines. DNA copy number profiles for chromosome region 2p25.3-2q14.3 (approximately every 1.3 Mbps) containing the *MYCN *gene were derived from 96 oligonucleotide primers. All 12 neuroblastoma-derived cell lines were examined, but only the SH-SY5Y, TNB-1, NGP and SK-N-BE lines are displayed. Horizontal axis indicates each locus from 2pter (bp), and vertical axis shows test/reference log_2 _fluorescence ratio. The *MYCN *loci are indicated by black circles. **C. **CGP high-resolution analysis (approximately every 48 kbps) in neuroblastoma cell lines. DNA copy number profiles for chromosome 2p24.2-2p24.3 containing the *MYCN *gene were derived from 72 oligonucleotide primers and represented 66 loci. All 12 neuroblastoma-derived cell lines were examined, but only NBL-S, SK-N-DZ, TGW and RTBM1 cell lines are displayed. Horizontal axis indicates each locus from 2pter, and vertical axis shows test/reference log_2 _fluorescence ratio. The arrow and black circles denote the *MYCN *loci. **D. **Summary of the amplified region surrounding the *MYCN *gene. The vertical axis indicates the amplified loci between 15.5 Mega (M) bp and 17.5 M bp from 2pter. The black bars denote the DNA amplification sites for each cell line. Regions where amplifications were detected in more than 2 spots of the CGP assay were defined as "amplification sites". The dashed line represents the loci of *NAG, DDX1, MYCN *and *FAM49A*.

Neuroblastoma is one of the most common pediatric solid tumors and accounts for 7–10% of all childhood cancers. The prognosis of patients varies according to stage, age and *MYCN *amplification status. The *MYCN *oncogene (located at 2p24.3) is particularly important. Since it was first described in 1983 [20] it has served a fundamental prognostic role for neuroblastoma patients. *MYCN *amplification has been detected in more than 80% of neuroblastoma cell lines [21], whereas other regions with high-level genomic amplification have rarely been observed. We verified *MYCN *amplification in ten neuroblastoma cell lines by Southern hybridization. This gene was amplified in all ten cell lines except SH-SY5Y and NBL-S (Fig. 4A).

Initially, the CGP assay was performed on all the neuroblastoma cell lines using 96 primers located approximately every 1.3 Mbps, spanning 2p25.3 to 2q14.3 and containing the *MYCN *gene. In all the lines except SH-SY5Y and NBL-S, CGP detected the amplified *MYCN *gene (Fig. 4B) in agreement with the Southern blot analysis. Focusing on the region adjacent to the *MYCN *gene locus, we generated 72 primers approximately every 48 kbps between 2p24.3 and 2p24.2. These primer sequences are supplied as the additional data file 2. Each cell line except SH-SY5Y and NBL-S had an amplified region that was detected by the CGP assay. For instance, NBL-S cells showed no amplification in this region, whereas amplification was observed at 2p24.3 in SK-N-DZ and RTBM1 (15,501,688-16,300,557 bp from the short arm telomeric end (2pter) of chromosome 2 and 15,890,708-16,320,323 bp from 2pter, respectively), including amplification of the *MYCN *gene (Fig. 4C). *MYCN *amplification was detected in the TGW cell line, but it was accompanied by discontinuities between the top of the *MYCN *gene and the middle of 2p24.2 (16,032,021 bp and 17,263,397 bp from 2pter). The amplified region in the vicinity of the *MYCN *gene is summarized in Fig. 4D. Only the neuroblastoma-amplified gene (*NAG*), DEAD (Asp-Glu-Ala-Asp) box polypeptide 1 (*DDX1*), *MYCN *and hypothetical protein LOC81553 (*FAM49A*) are present in this region between 15.5 × 10^6 ^bp and 17.5 × 10^6 ^bp from 2pter [22]. *NAG *and *DDX1 *are reported to be co-amplified with the *MYCN *gene in neuroblastoma [23], but amplification of *DDX1 *and/or *NAG *has no additional adverse effect on prognosis in this disease [24,25].

### Comparing CGP to an oligonucleotide array

**Figure 5:**
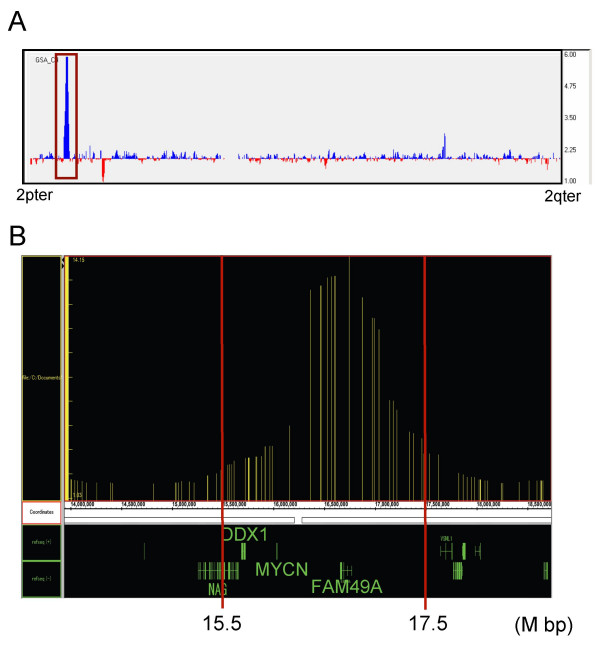
Detection of *MYCN *gene amplification in TGW cell line using oligonucelotide arrays. **A. **DNA copy number changes on chromosome 2. The horizontal axis indicates each locus from 2pter to 2qter, and the vertical axis shows estimated copy number. Gains or losses in DNA copy number are indicated by the blue and red bars, respectively. The brown box depicts the amplified region in chromosome 2 and is expanded in B. **B. **The region adjacent to the *MYCN *gene locus highlights chromosomal changes (the brown box region in Fig.5A). The Integrated Genome Browser (IGB) was used for visualization of the data. The horizontal axis indicates chromosome locus, and the vertical axis shows estimated copy number. The two red lines indicate 15.5 M bp and 17.5 M bp, respectively, as in Fig. 4D.

To compare CGP with microarrays, we used both methods to measure DNA copy numbers on chromosome 2 in TGW cells. We chose the Affymetrix GeneChip 50K Hind array, which has a mean marker distance of 50 kb and has a resolution equal to the previous CGP experiments (Fig. 4C). An intensely amplified region was observed on the short arm of chromosome 2 (Fig. 5A, B). However, additional DNA aberrations were detected only by CGP (Fig. 4C, TGW). In addition, no specific amplification of the *MYCN *gene was detected by the Affymetrix array because of a lack of probes in the *MYCN *region.

## Discussion

In this study we describe a novel PCR-based method for detecting changes in genomic DNA copy number. Although techniques have been described that use ligation of synthetic oligonucleotides for genomic amplification and RNA quantification, CGP is the first to use them for quantifying genomic DNA [26]. In the CGP assay we used the PstI restriction enzyme, which recognizes the 6 bp sequence CTGCAG, to fractionate the DNA. The high prevalence of PstI sites in genomic DNA offers a mapping resolution of approximately 4^6 ^= 4096 bps, which is significantly greater than FISH-based CGH (~20 Mb). PstI could be substituted by another enzyme such as SacI, HindIII or XhoI (which are unaffected by DNA modification) if no PstI sites are present near the gene of interest.

CGP is capable of detecting small copy number differences such as XX versus XY, XXX, XXXX or XXXXX. The mean relative ratio values for chromosome X loci were 0.69 for XY/XX, 1.0 for XX/XX, 1.37 for XXX/XX, 1.62 for XXXX/XX and 2.08 for XXXXX/XX, as shown in Fig. 2D. These data are comparable to those obtained with array based-CGH [3,4].

As a model for the study of gene amplification in human cancer, we focused on *MYCN *gene copy alterations in neuroblastoma-derived cell lines. We used the CGP assay to detect gene amplification in each cell line at approximately every 1.3 Mbps in the vicinity of the *MYCN *gene, and observed *MYCN *gene amplification in all but two of the lines, SH-SY5Y and NBL-S. Furthermore, examination of the region adjacent to the *MYCN *gene locus at approximately every 48 kbps revealed that these cell lines had a diversity of small region amplifications between 15.5 × 10^6 ^bp and 17.5 × 10^6 ^bp from 2pter (Fig. 4D). A recently updated gene assembly database showed that *NAG*, *DDX1*, *MYCN *and *FAM49A *map to this same region on chromosome 2. These genes have previously been shown to be amplified in cancer, but it is also possible to use CGP to focus on or identify additional genes involved in cancer or other diseases.

Owing to its robustness, PCR offers a reliable alternative to DNA hybridization-based techniques. For example, quantitative PCR is routinely used to confirm results obtained by DNA microarrays. Thus, although the current versions of microarray-based techniques are sound, it is desirable to have an alternative PCR-based technique to confirm the results. Real-time PCR has been used for this purpose but tedious calibration experiments make it difficult to use for high-throughput applications [8]. In CGH, two genomic sources are labeled with two different fluorescent dyes and hybridized in a single reaction vessel. Because these labeled DNAs are competitively hybridized to chromosomal DNA or spotted BAC DNA, the ratio of the two dyes indicates quantitative differences in the particular locus of the genome. In CGP, two DNA sources are differentiated after competitive PCR by the size of the adaptors. In principle, both techniques use a competitive reaction between two different genomic DNA sources. As indicated by experiments with various numbers of X chromosomes, the CGP technique provided the desirable level of quantification. It should be noted that both techniques are limited in dynamic range: for example, the dynamic range of CGP would be up to about 20-fold as estimated from the results of adaptor-tagged competitive PCR [27], a similar technique for quantifying RNA.

CGP lends itself to applications in several specific cases. Because the throughput of the technique is moderate, it is easy to profile chromosomal gains or losses at the resolution of conventional CGH. Microarrays would be the method of choice for profiling the entire genome, and CGP may be used to complement it. In this study, CGP detected DNA discontinuities that were not found with a microarray. This suggests that commercial arrays may miss chromosomal alterations because of gaps between probes. If a particular chromosomal region is important for a piece of research, more data points should be added, and CGP can be a useful tool for such purposes. The main merit of CGP is the ease with which any locus in the human (or other) genome may be analyzed simply by making a single PCR primer. As shown by the example of *MYCN *in neuroblastoma, high-resolution mapping of a particular genome locus is one of the major applications of CGP. Such high-resolution mapping can be achieved with less cost and effort than hybridization-based techniques. In addition, the robustness of PCR would be advantageous in cases with limited DNA sources such as those obtained by laser micro-beam dissection, a promising technique for cancer research.

The above applications of CGP are for research purposes. In the near future, it will be important to analyze the loss or gain of chromosomal loci for diagnosis. In such cases, CGP will be a rapid and accurate method and a strong candidate for clinical applications.

## Conclusion

CGP is a moderate throughput PCR-based technique for analyzing changes in genomic copy number. Because CGP can measure any genomic locus just by supplying PCR primers, it is especially useful for detailed analysis of a genomic region of interest at a higher resolution than commercial CGH arrays.

## Methods

### Genomic DNA

Blood samples were obtained from 10 healthy male and 10 female Japanese volunteers with informed consent. Equal quantities of genomic DNA were prepared from each blood sample as previously described [28] and the samples from each gender were mixed (Mmix or Fmix) to avoid variations in genomic copy number in the control samples [29,30]. Genomic DNAs from human cell lines were also used. Genomic DNAs from the following cell lines were obtained from the Coriell Cell Repository for Medical Research (Camden, NJ, USA): 47, XXX (repository no. NA04626), 48, XXXX (repository no. NA01416), and 49, XXXXX (repository no. NA06061). Genomic DNAs from neuroblastoma-derived cell lines (SH-SY5Y, NBL-S, NGP, CHP134, SMS-SAN, SK-N-BE, SK-N-DZ, TGW, LA-N-5, RTBM1, TNB-1 and GOTO) were obtained from the Chiba Cancer Center Research Institute (Chiba, Japan). For each reaction (locus) of the CGP assay, 1 ng of each genomic DNA test sample was used.

### Design of PCR primers and adaptors

To measure the genome copy number, locus-specific primers were designed. There were 132 primers for chromosome X, 60 primers for chromosome 17 and 96 primers for chromosome 2, all of which were 20-mer oligonucleotides. The primers consisted of 18 bases of genomic DNA sequence from a region that was 15–65 bases upstream or downstream of a PstI site, and GT nucleotides were added to the 5' end of each primer so that Taq DNA polymerase would add an "A" to the end of the strand extending from the adaptor primer [31]. Addition of GT allows us to omit the trimming step by T4 DNA polymerase before measuring the quantity of fragments. All primers used in this study were custom-made by Hokkaido System Science (Hokkaido, Japan). The sequences of the adaptors (designated PT1 to PT3) and the fluorescent dye-labeled adaptor primer are shown in Table 1. The size differences between the adaptors were three bases, the minimum distance required for complete separation of adjacent peaks. 6-Carboxyfluorescein (FAM) was used to label the adaptor primer.

**Table 1:** Sequences of adaptors and the adaptor primer.

Name	Oligomer	Sequences
**PT adaptors**		
PT1		
	bPT1L	5'-biotin-**GTACATATTGTCGTTAGAACGCG**TCAATCCATACTTGCA-3'
	PT1S	5'-AGTATGGATTGA**CGCGTTCTAACGACAATATGTAC**-3'
PT2		
	bPT2L	5'-biotin-**GTACATATTGTCGTTAGAACGCG**TACTCAATCCATACTTGCA-3'
	PT2S	5'-AGTATGGATTGAGTA**CGCGTTCTAACGACAATATGTAC**-3'
PT3		
	bPT3L	5'-biotin-**GTACATATTGTCGTTAGAACGCG**CTATACTCAATCCATACTTGCA-3'
	PT3S	5'-AGTATGGATTGAGTATAG**CGCGTTCTAACGACAATATGTAC**-3'

**Adaptor primer**		
C1S-FAM		5'-6FAM -GTACATATTGTCGTTAGAACGC-3'

Each adaptor consists of a pair of oligomers designated S and L. The three adaptors shared a common sequence (bold) and had a cohesive end complementary to the PstI restriction site (underlined). Biotin was added to the 5' ends of the longer oligomers for the subsequent purification step, and 6FAM was used to label the C1S adaptor primer, which was complementary to the shared sequence of the three adaptors.

### CGP assay and data processing

In the CGP assay, 1 μg of each genomic DNA was treated with 15U of the restriction enzyme PstI (TaKaRa, Kyoto, Japan) in a 20 μl reaction volume. The enzyme was inactivated at 70°C for 20 min, then 7.6 pmol of a 5'-biotinylated adaptor (PT1, PT2 or PT3 adaptor) was added to a 4 μl aliquot of the PstI-digested DNA (test or reference) in a 10 μl volume containing 1 × ligation buffer and 3U T4 DNA ligase (Promega, Madison, USA), and incubated at 16°C for more than 12 hours. After the enzyme has been heat-inactivated at 70°C for 20 min, the three genomic DNA samples were mixed as follows: 4 μl PT1, 4 μl PT2 and 2 μl PT3. The DNA mixture was purified twice on silica gel columns (QIAGEN GmbH, Germany) to remove enzymes, salt and short fragments such as unligated adaptors. The final 50 μl volume of DNA was treated with 20U of the restriction enzyme MboI (TaKaRa, Kyoto, Japan) in a 100 μl reaction mixture at 37°C for 20 min, followed by the addition of 25 μl 5 M NaCl. Fifty microliters of streptavidin-coated paramagnetic beads (1 mg/ml, Dynal Biotech, USA), which were initially washed with 50 μl of 1 × B&W buffer [10 mM TrisHCl (pH 7.5), 1 M NaCl and 1 mM EDTA], were added to the mixture, incubated for 20 min at room temperature, then washed three times with distilled water (DW) to remove the genomic DNA fragments cleaved by MboI. DW was added to adjust the volume to 200 μl. Each set of PCR premixtures consisted of 110 μl 10 × PCR buffer, 13.2 μl 20 mM dNTP mixture, 1320 pmol C1S-FAM primer and 150U AmpliTaq Gold (Applied Biosystems, CA USA) in a final volume of 800 μl. For a single round of PCR reactions, 200 μl of the master DNA template mixture was combined with 800 μl of the PCR premixture, and a 9 μl aliquot of this solution was added to each well of a 96-well microtiter plate. One microliter of each 10 pmol/μl locus-specific primer solution was then added to each well. The PCR cycling program was as follows: 95°C for 10 min followed by five cycles of 94°C for 30 s, 65°C for 30 s and 72°C for 45 s; a second set of five cycles of 94°C for 30 s, 60°C for 30 s and 72°C for 45 s; a third set of 10 cycles of 94°C for 30 s, 55°C for 30 s and 72°C for 45 s; and finally 30 cycles of 94°C for 30 s, 50°C for 30 s and 72°C for 45 s, followed by 72°C for 30 min. The PCR products were loaded and separated by the automated sequencer ABI 3730 (Applied Biosystems, CA USA), and the fluorescent intensity of each peak was recorded. We applied a single sample in one capillary, and the running time of electrophoresis was less than 30 minutes: we processed more than 1,000 samples per day.

The shortest and longest adaptors (PT1 and PT3) were used for control references (Fmix DNA) in equivalents of one and of one half, respectively, while the medium-length adaptor (PT2) was used for the test sample in one equivalent (Fig. 1). Therefore, the expected DNA copy number in the test sample was:

Test/reference fluorescence ratio = 2*x*_PT2_/(*x*_PT1_+2*x*_PT3_)

Here, *x*_PT1_, *x*_PT2 _or *x*_PT3 _are the heights of the peaks containing locus-specific DNA ligated to PT1, PT2 or PT3, respectively (Fig. 1). If the raw peak height of PT1 was lower than 1,000 in fluorescence intensity, the data were omitted.

### Southern hybridization to detect MYCN amplification

Five micrograms of high molecular weight genomic DNA prepared from various neuroblastoma-derived cell lines were digested completely with EcoRI, size-fractionated on 1% agarose gels, transferred to nylon membrane filters and immobilized by UV irradiation. The hybridization probe was prepared from gel-purified MYCN cDNA fragments, which were radio-labeled with [α-^32^P] dCTP by the random-priming procedure. Hybridization was performed overnight at 65°C in a solution containing 1 M NaCl, 1% N-lauroyl sarcosine, 7.5% dextran sulfate, 100 μg/ml heat-denatured salmon sperm DNA and the radiolabeled probe DNA. After hybridization, the membrane filters were washed twice with 2 × SSC/0.1% N-lauroyl sarcosine at room temperature followed by one wash with 0.1 × SSC/0.1% N-lauroyl sarcosine at 65°C and exposed to X-ray film with an intensifying screen at -70°C.

### Oligonucleotide array analysis

The Affymetrix Gene Chip Human Mapping 50 K Hind Array (Santa Clara, CA) was used for all experiments following the GeneChip Mapping 50 K protocols. Fmix DNA served as a control. The Affymetrix Chromosome Copy Number Anaylysis Tool (CNAT) 4.0 was used for the data analysis.

## Authors' contributions

KK conceived and designed the study. KIK designed the detail of the technique, and did the most of the experiments. KM, SS and SK assisted with the experiments. YN and AN prepared the neuroblastoma cell lines and their genomic DNA. KIK wrote the paper under the supervision of KK. All authors read and approved the manuscript.

## Supplementary Material

Additional file 1 The primer sequences for the whole genome. Click here for file

Additional file 2 Primers for figure 4c Click here for file
